# Effect of a Nutritional Support System (Diet and Supplements) for Improving Gross Motor Function in Cerebral Palsy: An Exploratory Randomized Controlled Clinical Trial

**DOI:** 10.3390/foods9101449

**Published:** 2020-10-13

**Authors:** Fernando Leal-Martínez, Denise Franco, Andrea Peña-Ruiz, Fabiola Castro-Silva, Andrea A. Escudero-Espinosa, Oscar G. Rolón-Lacarrier, Mardia López-Alarcón, Ximena De León, Mariana Linares-Eslava, Antonio Ibarra

**Affiliations:** 1 Centro de Investigación en Ciencias de la Salud (CICSA), FCS, Universidad Anáhuac México Norte, Huixquilucan 52786, Mexico; ferman5@hotmail.com (F.L.-M.); iantonio65@yahoo.com (D.F.); iantonio65@gmail.com (A.P.-R.); ceciggg73@gmail.com (X.D.L.); liesmari@hotmail.com (M.L.-E.); 2 Departamento de Terapia Física, Centro de Rehabilitación e Inclusión Infantil Teletón (CRIT), Tlalnepantla de Baz 54010, Mexico; juan.ibarra@softhealth.com.mx (F.C.-S.); andyibarra03@gmail.com (A.A.E.-E.); 3 Departamento de Investigación y Enseñanza, Centro de Rehabilitación e Inclusión Infantil Teletón (CRIT), Tlalnepantla de Baz 54010, Mexico; edyibarra05@gmail.com; 4 Unidad de Investigación Médica en Nutrición, Hospital de Pediatría CMN siglo XXI, Ciudad de Mexico 06720, Mexico; mardyalo@hotmail.com

**Keywords:** child, cerebral palsy, diet modification, motor development delay, nutrition disorder, nutritional support

## Abstract

Background: Most patients with cerebral palsy (CP) do not respond to physical therapy due to deterioration in their nutritional status, secondary to gastrointestinal disorders and the catabolic state of the disease itself. However, basic treatments only contemplate the energy requirements and do not consider supplementation with glutamine, zinc, selenium, colecalciferol, spirulina, omega 3 or even vegetal proteins. Objective: In this study, we determined the effect of using a nutritional support system (NSS): diet and supplements, on the gross motor function in children with CP with spastic diparesic and Gross Motor Function Classification System III (GMFCS III). Methods: An exploratory study was performed. Thirty patients (from 4 to 12 years old) were randomly assigned to: (1) dietary surveillance (FG), (2) deworming and WHO diet (CG), or (3) deworming and the NSS (IG). Gross motor function was evaluated using the gross motor function measure (GMFM) scale. Results: The IG-treated group presented a significant improvement in standing and walking parameters analyzed in the GMFM compared with FG and CG groups. Fifty percent of the IG-treated patients managed to walk, while in the other groups, no patients were able to walk. Conclusions: The NSS used in the present work improves gross motor function and promotes walking in patients with CP.

## 1. Introduction

Cerebral palsy (CP) is the most common physical disability in childhood. Its prevalence worldwide and in developed countries is approximated to 2–2.5 cases per 1000 live births [1,2]. According to the Center for Disease Control (CDC), the cost per person of CP was estimated at around 921,000 dollars per year in the United States (2003), while the costs for general medical assistance amounted to 11,500 million dollars [3]. CP is a permanent movement disorder characterized by a persistent postural tone, causing limitation of activity. This is attributed to non-progressive damage on a developing and immature brain that is originated in the fetal, perinatal period (greater percentage), or first years of life. CP is accompanied by alterations in sensation, cognition, communication, perception, spasticity, seizures, disorders in swallowing, and malnutrition [4].

Traditional treatment for patients with CP generates a recovery of 2% [5] per year and is based on rehabilitation, botulinum toxin therapy, general care, and in the case of malnutrition or disorders in swallowing, nutritional support which is based on the adaptation of Krick to the Schofield formula [5,6]. Nevertheless, there are no specific recommendations for dietary intake in patients with CP. Moreover, the literature information about nutritional support for improving neurological function is scarce. That is why CP continues to be the focus of numerous investigations. In line with this, a novel strategy that includes nutrition and neurological remodeling support could be of valuable use for patients with CP since, at the same time that it nourishes, it significantly impacts the neurological recovery of the patient, especially motor function. Gross motor dysfunction is one of the main affectations in children with CP [7], and it is associated with spasticity, which reflects the damage in the Pyramidal System. This movement-alteration is also strongly associated with malnutrition in patients with CP [8]. Currently, the use of functional foods, supplements or even the administration of probiotics or prebiotics as a therapeutic strategy has become a very important research area [9]. In this field, numerous studies have tested—separately—the effect of some nutrients in the central nervous system (CNS); however, there are no investigations integrating these elements as a whole treatment in CP. Arginine, for instance, participates in the formation of nitric oxide (NO) which is associated with neuronal regeneration and protection of the CNS [10]. Docosahexaenoic acid (DHA) and sphingosine 1 phosphate (S1P) prevent the early death of newly generated neurons [11]. Omega 3 polyunsaturated fatty acids (PUFAs n-3) participate in brain plasticity, neurogenesis, and memory and brain repair. Neuromuscular alterations after cerebral ischemia, improve after omega-3 treatment [12,13]. On the other hand, supplementation with probiotics has also been proposed as a therapeutic strategy for alleviating CNS pathologies [14]. Probiotics have been used to stimulate the production of neurotransmitters, memory, neuroregeneration, and also for correcting malabsorption [15]. In this work, probiotics were used for the latter purpose.

In order to provide patients with CP with the best therapeutic strategy, it is also important to consider the use of several metabolic rescue pathways to produce energy, including lactate and alanine cycles, where glutamine and glutamic acid are the main substrates [16]. Deficiencies in plasma concentrations of iron, folate, niacin, calcium, vitamins D and E, zinc, selenium, and proteins have been in reported in children with CP, even in children who were being supplemented. Therefore, supplementation of some nutrients and proteins should also be considered [17,18,19]. Regarding protein supplementation, it is relevant to mention that protein plant supplements such as Spirulina Maxima, in addition to providing a high protein intake, produce compounds such as PUFAs and are also a good source of vitamins [20].

Finally, it is relevant to consider that 3500 million people in the world are parasitized and of them, the majority are children [21]. This, and other factors such as the catabolic increment observed in neurologic patients, difficulties for feeding mechanics, gastrointestinal alterations, and the use of drugs that compete with the transport, absorption, or metabolism of nutrients, increases the risk of malnutrition in children with CP [22,23]. Therefore, parasitosis makes malnutrition an even more significant problem in this group of people.

It can be said, therefore, that there are several factors that need to be addressed in order to establish the best therapeutic strategy for CP. At the moment, there is no scientific evidence of an integrative nutritional support. That is why, in the present study, we tested an integral food system—the nutritional support system (NSS)—that includes nutrition, metabolic support, deworming, and neurological remodeling support.

## 2. Materials and Methods

The exploratory study was designed as a controlled clinical trial which was randomized, and blinded; children with CP with spastic diparesis and GMFCS III were enrolled in this study (during a period of 3 years) and treated at the Children’s Telethon Rehabilitation Center (CRIT) in Tlalnepantla Estado de México. The duration of the study was 13 weeks for each participant.

The trial complied with the principles of the Declaration of Helsinki and the Mexican norm NOM-012-SSA3-2012 for scientific research in humans. All procedures were approved by the Committee of Research of the Faculty of Health Sciences of the Universidad Anáhuac México Campus Norte with the number 2014/03001. The parent or guardian and the patient (in the event that they could sign) signed the informed consent letter voluntarily. The Clinical Trials identifier of this study is: NCT03933709.

### 2.1. Participants

**Figure 1:**
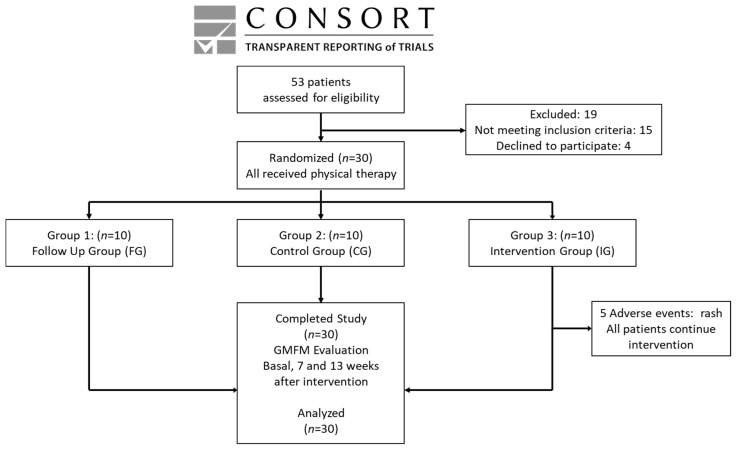
Consort, transparent reporting of trials.

Fifty-three children were interviewed and, from this group, thirty met the inclusion criteria (see Figure 1).

Inclusion criteria: Patients with CP with spastic diparesis and GMFCS III (these patients demonstrate elevated functionality in the categories of lying/rolling, sitting, and crawling), of both genders aged between 4 and 12 years old, who had support from a full-time caregiver, and who were able to feed orally.

Non-inclusion criteria: Patients with CP presenting another catabolic disease which could increase the risk of malnutrition (renal, cardiovascular, pulmonary, hepatic, or immunological disease), and those presenting infections or receiving antibiotic treatment at least 15 days before starting the study; patients who had received therapy with botulinum toxin or muscle relaxants in the last 6 months; patients with CP presenting severe gastroesophageal reflux or with any type of surgery performed in the last 9 months; and patients walking by themselves.

### 2.2. Group Assignments

Before recruiting patients, thirty numbers (from 1 to 30) were placed in a tombola. Afterwards, each number was randomly selected from the tombola and sequentially allocated to one of three groups, until reaching 10 numbers per group. Each number represented the time the patient was to be enrolled. This way, once initiated the recruitment, the first patient was assigned number one; the second was number two, and so on. In this fashion, each patient was allocated (according to the time of enrollment) to the group to which, their number corresponded.

Afterwards, the patients were informed and trained according to their corresponding groups: (1) the Follow-up Group (FG, *n* = 10)—only their usual diet was monitored; (2) the Control Group (CG, *n* = 10) was dewormed and received the nutritional therapy recommended by the WHO [23]; (3) the Intervention Group (IG, *n* = 10) was dewormed and received the NSS. As parasitosis is a common variable that could affect the absorption of nutrients among the groups and, as a part of our hypothesis, is related to the positive effect that deworming could have on the absorption of supplements, we decided to contrast the IG group versus a dewormed (CG) group and a non-dewormed (FG) group.

All participants received Bobath physical therapy.

### 2.3. Procedures

Once the participants were selected, parents and children with CP were called to explain the protocol and to collect their informed consent forms. Upon entering the study, the nutritional history and gross motor function measure (GMFM) scale qualification was obtained and recorded from each patient. Thereafter, patients with CP were randomly allocated into the 3 groups (FG, CG, and IG). The energy calculation for the CG and IG groups was performed with the Krick formula (50% carbohydrates, 30% lipids, and 20% proteins).

The parents and/or caregivers of the participants were trained on the project, procedures, as well as feeding and supplementation schemes if required. They were also warned to avoid any comment on the treatment that the patient received. At the beginning of the treatment, the CG and IG groups were dewormed with nitazoxanide at a dosage of 7.5 mg/kg every 12 h for 3 days.

### 2.4. Diet, Shakes, and Supplementation

**Figure 2:**
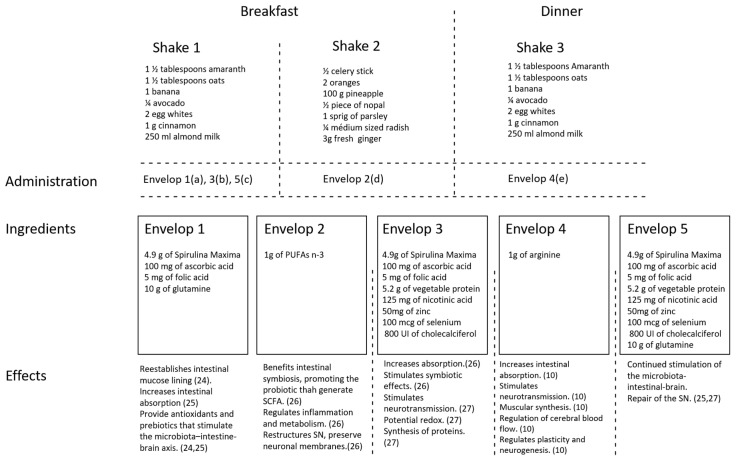
Schematic overview of ingredients, administration, and expected function of shakes and envelopes. (**a**) Administered only the first 10 days of therapy; (**b**) administered from day 11 until the end of week 6, after which it was suspended and substituted for envelope 5 (10 days), and then, it was retaken until the end of the intervention; (**c**) administered only during 10 days after the 6th week of therapy, after which envelope 3 was restarted; (**d**) administered throughout the intervention; (**e**) administered from day 8 until the end of intervention.

Our nutritional support system consists of a shake-based diet with functional ingredients, high levels of vegetables, fruits, cereals, root-vegetables, and fish. Additionally, it is supplemented with glutamine, arginine, folic acid, nicotinic acid, zinc, selenium, cholecalciferol, ascorbic acid, spirulina, vegetal protein, PUFAs n-3, and probiotics (Saccharomyces Boulardii; 200 mg every 12 h for 3 days in the basal period and at week 7, for correcting malabsorption). In order to ensure that patients reached the necessary caloric intake, they were instructed to take two shakes in the morning and one in the evening. Shakes were made at home by the parents and consumed immediately after preparation. A shake-based diet was chosen since it facilitates nutrient absorption in the intestine. Envelopes containing the ingredients for the shakes were provided to encourage consumption of the shakes and to make it easier to adhere to the therapy. The IG supplements were mixed in numbered envelopes and administered in three shakes: Shakes 1 and 3 contained amaranth (1½ tablespoon), oats (1½ tablespoon), 1/4 of a medium avocado, 1 medium-sized banana, egg whites (two), cinnamon (1 g) and almond milk (250 mL)—equivalent to 650 kcal, 14 g of protein, 11 g of lipids, and 58 g of carbohydrates. Shake 2 contained celery (1/2 of the stalk), oranges (two), pineapple (100 g), nopal (1/2 of a medium leaf), 1 sprig of parsley, radish (1/4 of a medium radish), and ginger (3 g) equivalent to 148 kcal, 3.5 g of protein, 3.5 g of lipids, and 36 g of carbohydrates and was to be drunk in the morning together with shake 1 (see Figure 2).The ingredients and administration of the envelopes were as follows: Envelope 1 contained 4.9 g of Spirulina Maxima, 100 mg ascorbic acid, 5 mg folic acid, and 10 g of glutamine. This envelope was to be added to shake 1 during the first 10 days of the intervention. Envelope 2 contained 1g PUFAs n-3 and was to be added to shake 2 which was given throughout the intervention. Envelope 3 contained 4.9 g of Spirulina Maxima, 100 mg ascorbic acid, 5 mg folic acid, 5.2 g vegetable protein, 125 mg nicotinic acid, 50 mg zinc, 100 mcg selenium and 800 UI cholecalciferol. This envelope was to be added to shake 1 from day 11 until the end of week 6, after which it was suspended for 10 days and substituted for envelope 5 and then to be retaken until the end of the intervention. Envelope 4 contained 1 g arginine and was to be added to shake 3 from day 8 until the end of the intervention. Envelope 5 contained the same ingredients as envelope 3 with an additional 10 g glutamine and was to be added to shake 1 from the start of week 7 for 10 days, after which envelope 3 was restarted. Ingredients in envelopes provide diverse beneficial effects [24,25,26,27] (see Figure 2). 

The total supplementation provides 287.28 kcal, 39.92 g of protein, 20 g of carbohydrates, and 4.96 g of lipids. The micronutrients that it provides are 3.81 g of fiber, 207.58 mg of sodium, 194 mg of calcium, 155 mg of phosphorus, 76 mg of magnesium, 6 mg of iron, 228.8 mg of potassium, 141.28 mg of zinc, 400 μg of selenium, 600 mg of vitamin A, 420 mg of vitamin C, 1600IU of vitamin D, 2.16 mg of vitamin E, 3.58 mg of vitamin B1, 0.76 mg of vitamin B2, 288.5 mg of vitamin B3, 8.81 mg of vitamin B5, 0.12 mg of vitamin B6, and 0.04 mg of vitamin B12.

### 2.5. Follow Up

The participants attended the CRIT twice a week to receive physical therapy and once a week for a clinical and nutritional review. In the nutritional review every week with the parents or caregivers, it was checked that the patient had no gastrointestinal problems and that they tolerated the oral route well. Food diaries were also reviewed, and patients handed over empty supplement envelopes to verify adherence. On days the patients attended physical therapy, they were supervised to ensure that they had breakfast, a mid-morning snack, and lunch.

### 2.6. GMFM Assessment

The GMFM scale was performed at baseline time and weeks 7 and 13 after intervention. The evaluators and the CRIT staff were the blinded aspects of the study as they did not have access to any information about the treatment each child was receiving. The patients and their families were not blinded, so they were warned to avoid any comment on the treatment that the patient received.

This scale assesses five general parameters: 1. Lying (decubitus) and rolling over (GMFAV), 2. Sitting (GMFB), 3. Crawling and kneeling (GMFC), 4. Standing (GMFD), 5. Walking (GMFE), and one final total item (GMFF). The scoring system consists of 88 items, and each one is valued based on the following criteria: 0 = No, 1 = start, 2 = Partially Complete, 3 = Complete, NE = Not evaluated [28]. The literature has reported a high convergent validity (0.91) of the GMFCS, on GMFM [29]. Robert Palisano, the author of the scale, attended the CRIT EDO MEX to carry out training to the evaluator of this study.

### 2.7. Statistical Methods

In order to know the distribution of the data, the test of Shapiro–Wilk was applied. Analysis of data was performed using the Mann Whitney U test or the Kruskal Wallis followed by the Dunn posthoc test. In order to calculate the size of the effect on standing and walking evaluations, we used Rosenthal’s r, an effect size test for data with non-normal distribution. This test can be used alongside Mann Whitney U-test results. The level of significance was considered as <0.05 in all cases.

### 2.8. Sample Size

As this investigation was an exploratory study, sample size was determined by the feasibility of recruitment. A sample of 10 children per group was established considering that the number of patients per year at CRIT—with the criteria required for the study—fluctuates between 10 and 15 patients. This sample size allows the detection of an effect size of 0.1 or larger. In order to reach the established sample, we required a three-year period.

## 3. Results

Fifty-three patients were interviewed. From this group, only thirty were recruited and studied from January 2015 until February 2018. The trial was stopped at this time to evaluate the preliminary results. The analysis of evaluations was made on the original assigned groups (three groups, 10 patients per group; see Figure 1). The demographic characteristics of the patients studied are summarized in Table 1.

As the adherence to the course of treatment was strictly supervised, more than 90% of patients complied with the intervention therapy. Each patient was requested to keep a food diary, which enabled us to establish their caloric intake per day. The average of kcal consumed at the start of the study in FG was 1292.5 kcal; in GC, 1495 kcal; and in IG, 1447.5 kcal. In week 13, the average kcal consumption was 1305 kcal in the FG group, 1113 kcal in CG, and 2898.8 kcal in IG.

### 3.1. Baseline Results

The baseline values of GMFM parameters (lying, sitting, crawling, standing, and walking) were not statistically different among the studied groups: lying (FG: 92.75 ± 3.44; CG: 93.34 ± 2.92; IG: 84.13 ± 8.87; mean ± SEM; *p* = 0.950; Kruskal Wallis followed by Dunn’s posthoc test), sitting (FG: 61.34 ± 5.24; CG: 74.15 ± 5.49; IG: 75.16 ± 8.09; *p* = 0.089), crawling (FG: 49.52 ± 5.56; CG: 47.38 ± 8.90; IG: 61.86 ± 7.79; *p* = 0.248), standing (FG: 13.44 ± 3.46; CG: 15.66 ± 3.25; IG: 16.79 ± 4.58; *p* = 0.666), walking (FG: 15.49 ± 1.51; CG: 18.05 ± 1.90; IG: 20.27 ± 3.24; *p* = 0.365).

### 3.2. Lying, Sitting, and Crawling Evaluations

Throughout the follow-up, lying and sitting parameters did not show any relevant difference among the groups. In the seventh week, the groups presented very similar values. Lying: FG: 95.69 ± 4; CG: 94.89 ± 7; IG: 83.92 ± 29; mean ± SEM; *p* = 0.982; Kruskal–Wallis followed by Dunn’s test. Sitting: FG: 73.83 ± 2; CG: 94.89 ± 7; IG: 83.92 ± 29; *p* = 0.114. In the 13th week, no significant difference was observed among the groups. Lying: FG: 97.63 ± 2; CG: 95.56 ± 5; IG: 89.2 ± 19; mean ± SEM; *p* = 0.960; Kruskal–Wallis followed by Dunn’s test. Sitting: FG: 77.33 ± 12; CG: 83.50 ± 15; IG: 90.60 ± 16; *p* = 0.082. With respect to crawling, there was not any significant difference at the seventh week: FG: 61.30 ± 19; CG: 57.29 ± 31; IG: 76.87 ± 28; *p* = 0.143, Kruskal–Wallis followed by Dunn’s test. Nevertheless, at the thirteenth week, evaluations presented a significant improvement in IG patients. Crawling: FG: 57.72 ± 17; CG: 54.38 ± 30; IG: 69.86 ± 26; *p* = 0.03, Kruskal–Wallis followed by Dunn’s test.

### 3.3. Standing and Walking Evaluations

**Figure 3:**
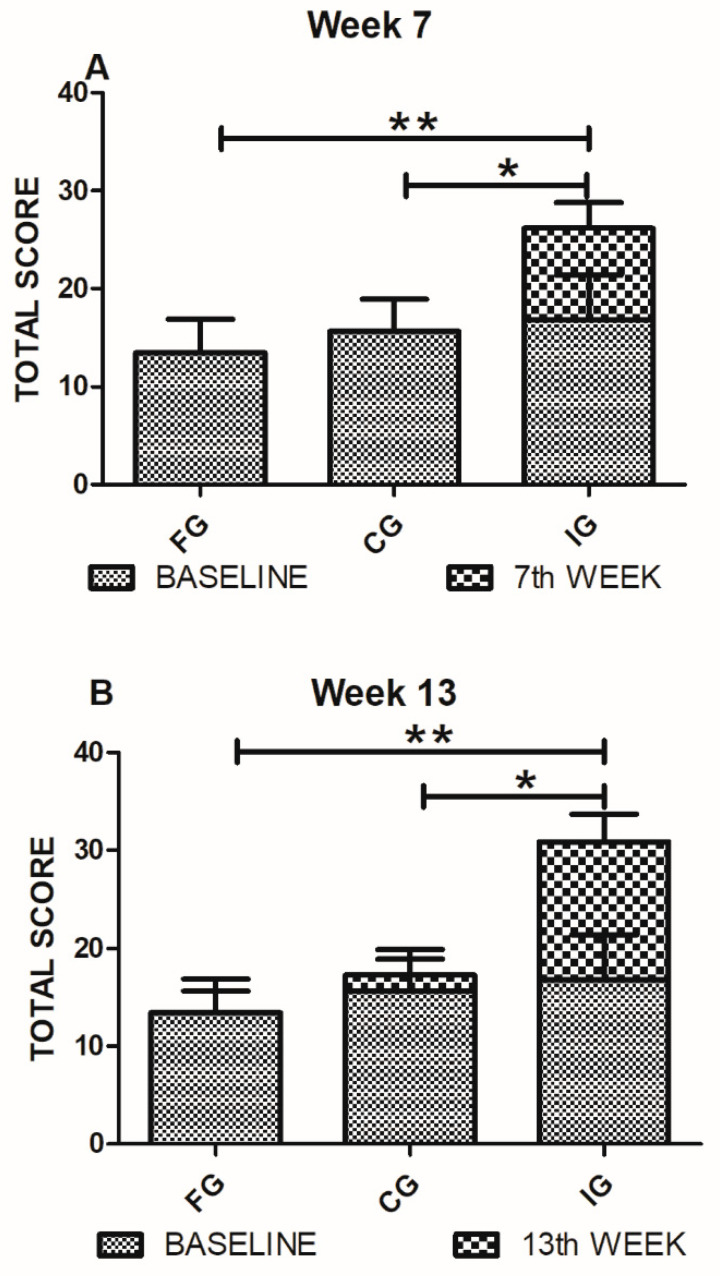
GMFD (standing) evaluations of FG, CG, and IG. (**A**) Standing evaluations on the seventh-week show a significant improvement of IG-patients compared to FG and CG ones. Analysis was performed by the Mann Whitney U test, * *p* = 0.003 ** *p* = 0.0004. (**B**) The standing evaluations at 13th-week show a significant improvement of the IG compared to the FG and CG groups. Analysis by the Mann Whitney U test, * *p* = 0.003 ** *p* = 0.0002. Bars represent mean ± SD of 10 patients. Baseline values are also shown.

Figure 3 shows the comparison of the FG, CG, and IG patients in the standing parameter at 7 and 13 weeks after the intervention. A total score was considered for these evaluations. Seven weeks after intervention, IG patients presented a significant improvement in standing (26.19 ± 8.2; mean ± SD) compared with FG (10.1 ± 4.9; *p* = 0.0004, Mann Whitney U test) and CG (12.98 ± 7.2; *p* = 0.003, Mann Whitney U test) groups. In order to strengthen these results, we calculated the Rosenthal’s r, a statistical assessment that evaluates the effect size (clinical efficacy of the treatment). Results showed a large size effect in the standing parameter of the IG group (r = 0.64). Thirteen weeks after intervention, the improvement continued being statistically different in IG-patients (30.90 ± 8.7) compared to FG (13.32 ± 7.4, *p* = 0.0002, Mann Whitney U test) and CG (17.29 ± 8.1, *p* = 0.003 Mann Whitney U test) groups. In this case, we also calculated Rosenthal’s r and found a large effect in the IG group (*r* = 0.61).

**Figure 4:**
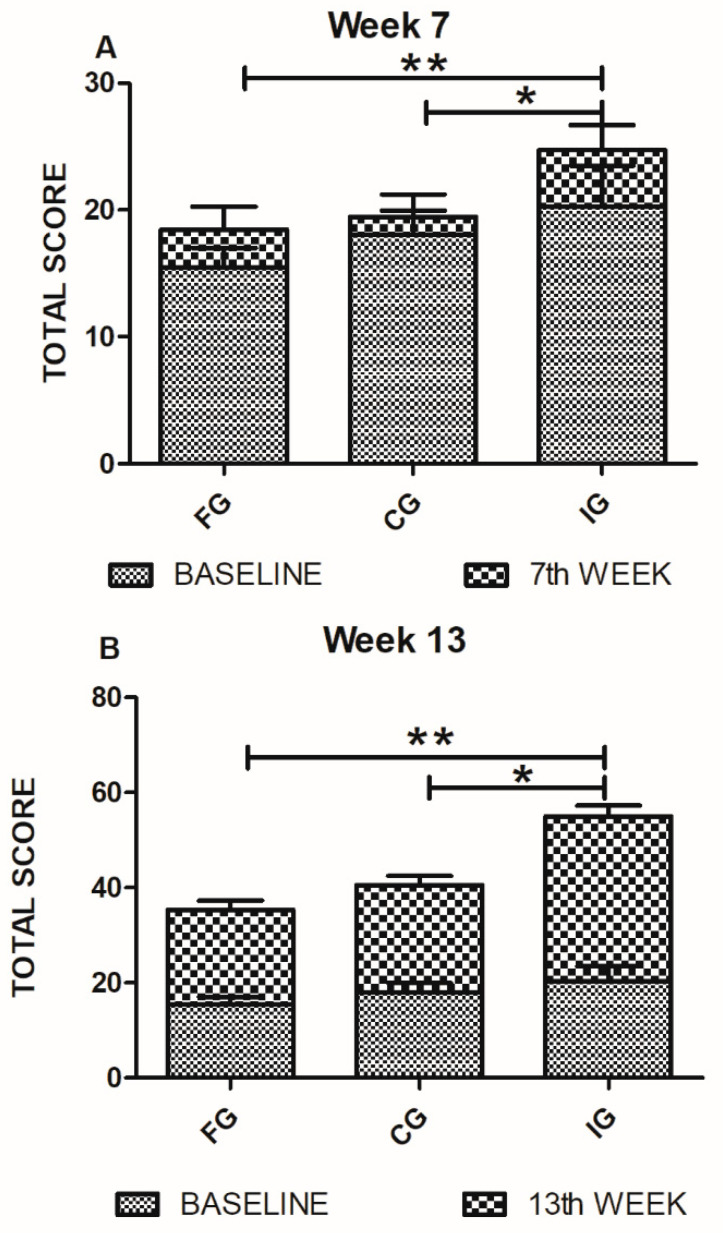
GMFE (walking) evaluations of FG, CG, and IG. (**A**) On the seventh week of evaluation, walking parameters showed a significant improvement in IG-patients. Analysis by the Mann Whitney U test. * *p* = 0.03 ** *p* = 0.01. (**B**) Walking evaluations on the 13th week showed a significant improvement of IG-patients compared with those of FG and CG. Analysis was performed using the Mann Whitney U test, * *p* = 0.001 ** *p* = 0.0003. Bars represent mean ± SD of 10 patients. Baseline values are also shown.

Figure 4 shows the comparison among FG, CG, and IG patients in walking parameter at 7 and 13 weeks after the intervention. Total score was considered. Seven weeks after intervention, there was a significant difference in walking improvement in IG-patients (24.65 ± 6.1) relative to FG (18.47 ± 5.7; *p* = 0.01, Mann Whitney U test) and CG (19.45 ± 5.6; *p* = 0.03, Mann Whitney U test) ones. Rosenthal’s r test showed a large effect size in the IG group (r = 0.37). After thirteen weeks, IG-patients continued presenting a significant improvement (34.68 ± 7.3) as compared to the FG (19.86 ± 6; *p* = 0.0003, Mann Whitney U test) and CG (22.51 ± 5.9; *p* = 0.001, Mann Whitney U test) patients. We also calculated Rosenthal’s r for this data set. Results showed a large size effect of treatment in IG-patients on walking parameter at thirteen weeks (r = 0.67). Finally, from this group of patients (IG), five managed to walk by themselves (with no support from anyone else). No patient from the FG or CG groups achieved walking independently.

### 3.4. Percentage of Evolution

**Figure 5:**
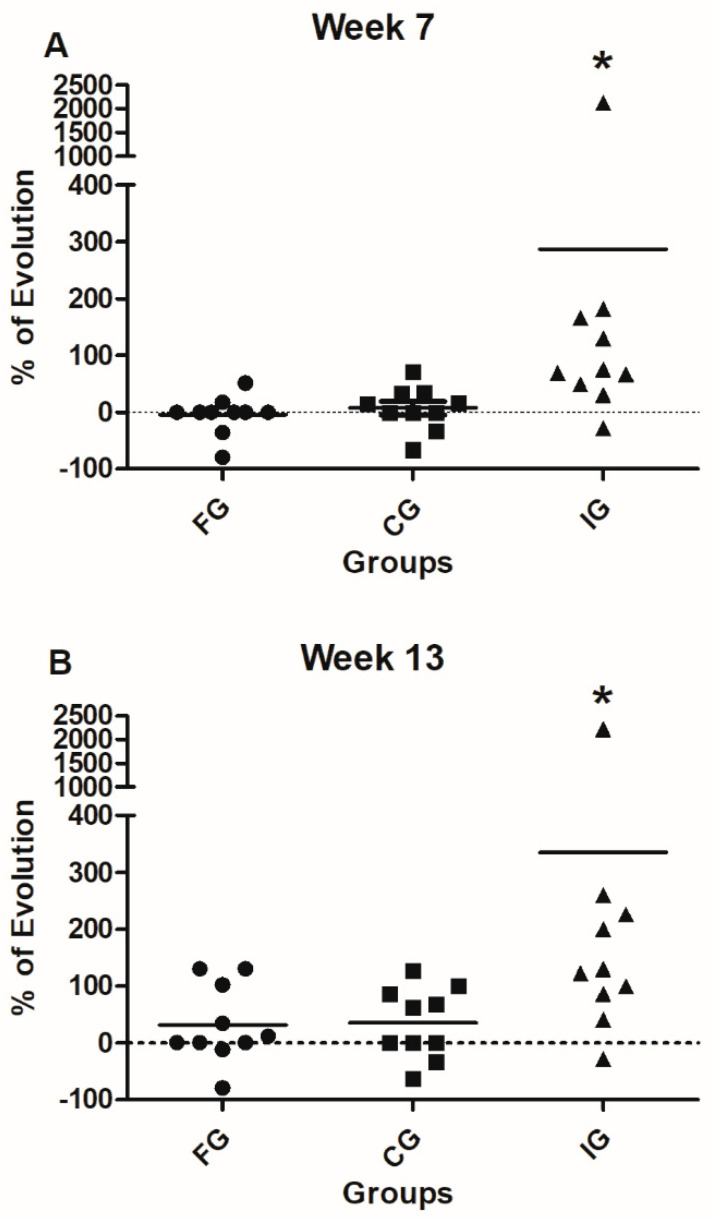
Percentage of evolution (GMFD, standing) of FG, CG, and IG. (**A**) The evolution from the baseline to the seventh week shows a significant improvement of standing ability in IG in comparison with the one observed in FG and CG patients. * *p* = 0.025, Kruskal Wallis test. (**B**) The evolution from the baseline towards the 13th week shows significant improvement in standing ability of IG compared to FG and CG patients. * *p* = 0.03, Kruskal Wallis test. Black line represents the mean of 10 patients.

Figure 5 shows the comparison of the percentage of evolution in standing among the studied groups. Percentage was calculated according to the formula: final corresponding value (7 or 13 weeks)/baseline value − 1 × 100). Therefore, we are reporting the percentage of evolution after 7 or 13 weeks of intervention. IG-patients showed a significant improvement in the percentage of evolution compared to both FG and CG patients (*p* = 0.025, Kruskall Wallis followed by Dunn’s posthoc test). The evolution towards the thirteenth week also revealed a significant difference in the improvement of IG-patients compared with FG and CG ones (*p* = 0.03, Kruskall Wallis followed by Dunn’s posthoc test).

**Figure 6:**
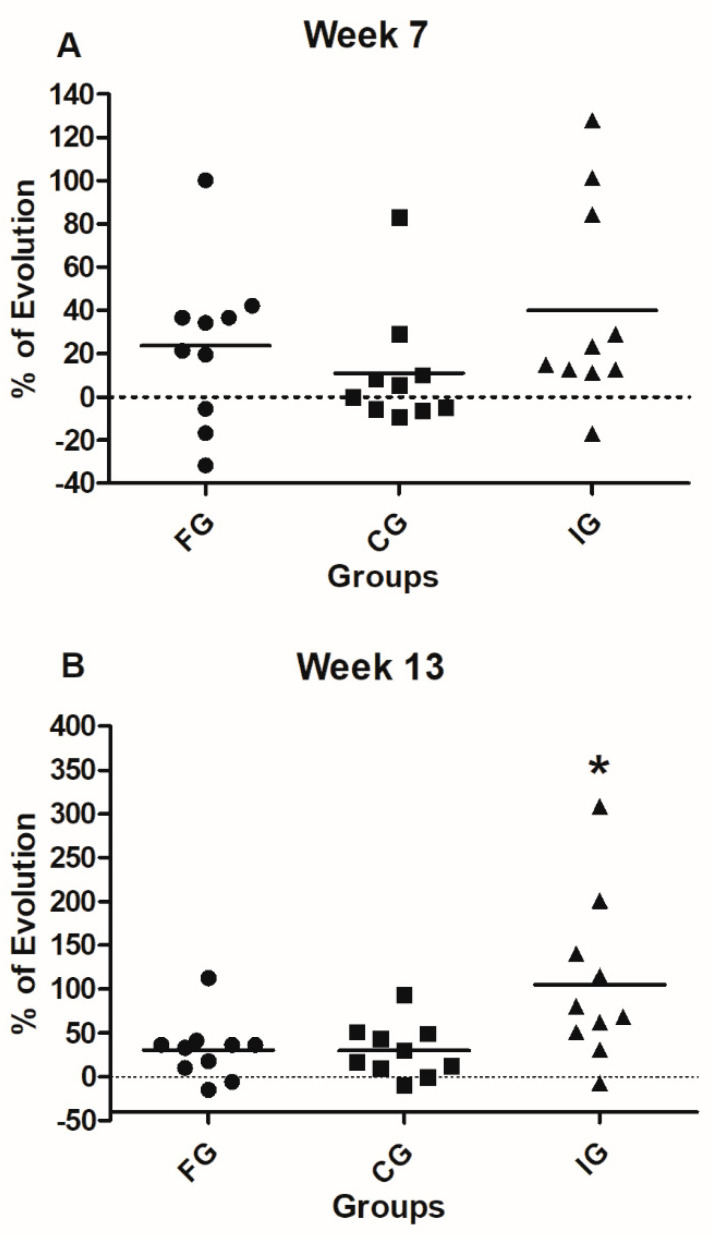
Percentages of evolution (GMFE, walking) of FG, CG, and IG. (**A**) The comparison of the percentages of evolution from the baseline to the seventh week does not show significant improvement in the progress of studied groups. Analysis was performed by using the Kruskal Wallis test. (**B**) The percentage of evolution from the baseline towards the 13th week shows significant improvement of the progress of IG compared to FG and CG patients., * *p* = 0.029, Kruskal Wallis test. Black line represents the mean of 10 patients.

When the walking parameter was analyzed, the evolution from the baseline to the seventh week did not present any significant advantage for any of the groups (Figure 6; *p* = 0.15, Kruskall Wallis followed by Dunn’s test). Nevertheless, in the evolution observed up to the thirteenth week, IG patients presented a significant improvement, compared with FG and CG patients (*p* = 0.03, Kruskall Wallis followed by Dunn’s test).

## 4. Discussion

In this work, we evaluated the effect of an NSS for improving motor function in patents with CP. Our findings showed that the NSS promoted a significant improvement in standing and walking parameters of the GMFM scale. Moreover, NSS-therapy caused half of the patients studied to walk by themselves. This is a relevant finding since the improvement in motor function was, in a shorter time, above the one observed in patients with CP undergoing only conventional therapy [5]. From the initial evaluation (7 weeks after the onset of therapy), the performance of IG-patients was better than the one observed in CG and FG groups, which presented a performance that was similar to that reported in the literature [30]. The improvement of IG-treated patients was evident thirteen weeks after therapy initiation. The results observed in IG-treated patients went beyond the ones reported before using only physical therapy (2–3% of annual advance) [5]. It is of relevance to mention that even though the baseline values of IG-patients were slightly higher, the difference among the groups was not statistically significant. In order to avoid any confusion on this matter, we performed an analysis of the percentage of improvement among the different groups (this analysis included the baseline values). Therefore, we are reporting the percentage of evolution after 7 or 13 weeks of intervention. The analysis confirmed that the results were not influenced by the baseline values.

The encouraging outcome of IG-treated patients could be due to the administration of diverse nutrients and supplements that are necessary in order to compensate the metabolic, nutritional, and neurological deficiencies presented by patients with CP.

Neurological patients regularly present an increased catabolism [31]; however, in some investigations, it is reported that some patients, especially those with CP, present a lower energy requirement than healthy patients although specific nutritional information, including proteins, is not known [32]. It was observed during this investigation that the energy consumption in the baseline of all the groups was around 1300 to 1400 kcal, and the nutriments intake increased in the IG patients around 2890 kcal per day on the 13th week, being the double of kcal intake in comparison with the other groups. It is likely that after deworming, supplementing, and consuming a specific diet, they were able to increase their calorie consumption. The NSS was the only different variable among the groups. Therefore, NSS could be—at least in part—the cause of this increment. Nevertheless, other factors—non-identified at this moment—could also be participating. Further studies should be designed to corroborate these results, find other possible factors, and to establish new nutritional recommendations in CP.

The observed improvements in standing and walking can be related to a reduction in spasticity. Changes were also observed in hand movements in the majority of IG patients, which resulted in the release of the thumb and pincer grip from the “claw hand”. Spasticity is related to a lesion in the Pyramidal System (PS); therefore, any improvement in these areas can be associated with a remodeling of the PS and thereby, of the CNS—in particular, in the areas of motor and premotor cortex as well as brainstem. This remodeling could be related with myelination, neurogenesis, promotion of release in neurotransmitters, or even the generation of ganglia [30]. The PS is made up of approximately one million fibers—mainly myelinated whose origins are in the primary motor and premotor cortex (80% of the fibers). Their function is to control voluntary movements, both fine and gross motor skills, in such a way that any alteration in these fibers can cause, amongst other alterations, spasticity [33].

Therefore, any therapy based on PS remodeling could be contributing to diminished spasticity and thereby, to improve fine and gross motor skills in patients with CP.

The induction or increment of plasticity phenomena, or even of regenerating neurons by providing substrates as nutrients, is extremely viable. A number of different nutrients, such as PUFAs n-3 (EPA and DHA), have been employed separately to stimulate plasticity and new neuronal cell bodies [30]. Nutrients such as zinc, ascorbic acid, spirulina, arginine, PUFAs n-3, and vitamin D have all been used in the repair of neuronal cell bodies and in neuronal regeneration [34,35]. Nevertheless, there is no scientific evidence from studies using an integrative nutritional support as a therapeutic strategy in CNS diseases. Moreover, investigations combining at least 3 or more nutrients are not available in the scientific literature.

On the other hand, it is known that the production of neurotransmitters such as serotonin and GABA can be increased using probiotics, nicotinic acid, cobalamine, pyridoxine, folates, zinc, ascorbic acid, and triptofane [36]. Regulating the production of serotonin and GABA can help to control spasticity and thus, fine and gross motor functions. The use of probiotics has become relevant in improving signaling from the intestine and favoring neurotransmission, metabolism, immunity, and inflammation through stimulating the microbiota-gut-brain axis [37].

Recently, studies on gut microbiota have become relevant in the treatment of various neurological disorders, including autism, alterations in memory, and neuronal regeneration [11]. It is also known that simultaneous supplementation of pro and prebiotics, such as inuline and other nutrients such as glutamine, induce the generation of short chain fatty acids which modulate inflammation and favor regeneration of the nervous system [38] as well as reestablishing enterocytes and muscular synthesis [39].

Finally, studies in mice showed that glutamine together with probiotics inhibit nitric oxide, reducing levels of proinflammatory factors such as TNF alfa, IL6, IL8, and reactive oxygen species (ROS) [40]. The evidence demonstrates that combining probiotics with other nutrients to create symbiosis induces positive effects on immunity, reestablishing mucose and the nervous system.

Although the results of this investigation are encouraging, it is important to consider the constraints of the study. Perhaps the main limitation of this work is the number of evaluated patients. In the present investigation, we could not gather more than 10 individuals per group. This was mainly because the patients did not meet the inclusion criteria. The use of other therapeutic alternatives (i.e., botulinum toxin or muscle relaxants) is very common in patients with CP, and this did not allow us to include them into the study. Further research needs to be undertaken in a larger sample to support the preliminary findings of this study. Additionally, we will implement evaluations such as anthropometry, electromyography, and gait and movement analysis.

On the other hand, it is important to carry out this investigation in a research center with a controlled system, where children and their caregivers could coexist closely during the 13 weeks of the protocol. Finally, despite the convincing clinical findings, it is important to complement further research with sensitive analytical methods to evaluate neuroregeneration or neurogenesis.

Regardless of all these constraints, our findings deserve to be considered for future investigations, since they are the result of a well-controlled and designed study, which, by nature, can be replicated and carried out in other populations. Additionally, the results of this study indicate a positive relationship between NSS and gross motor skills in CP. This means that children with CP who have high-energy supplementary foods, combined with micronutrients in special doses, will further improve gross motor skills. Ati et al. [41] reported similar observations: they found a significant relationship between nutritional status and gross motor children’s development. Finally, it is worth mentioning that in this investigation, nutrition was used as a stimulus to repair the nervous system—specifically, the pyramidal system. This is an innovative strategy with no previous similar studies. This exploratory investigation managed to induce better gross motor recovery in a shorter period of time as compared to conventional treatment.

Future studies should be directed to expand and confirm the results of this study.

## 5. Conclusions

Together, the results of this investigation provide insights into the positive effect of NSS on CP. We speculate that the observed results are mainly a consequence of a significant reduction in spasticity, which could be the result, at least in part, of a PS remodeling. In this scenario, the effect of all of the NSS components is crucial; however, we consider that the participation of PUFAs, zinc, ascorbic acid, spirulina, or arginine could be critical to repair neural function.

## Figures and Tables

**Table 1:** Clinical characterization of patients with cerebral palsy (CP).

Group	Gender	Age (Years)	GMFD	GMFE
		7.2 → 1.9	Basal Score	Basal Score
FG	F	9.6	7.7	11.4
FG	F	4.8	7.7	14.2
FG	M	5.7	7.7	18.3
FG	M	4.5	17.7	18.3
FG	M	6.8	5.1	18.3
FG	M	7.5	7.7	8.8
FG	F	5.1	27.7	25.0
FG	F	9.3	7.7	11.1
FG	M	5.5	37.7	16.7
FG	F	6.3	7.7	12.8
CG	M	9.8	23.1	16.7
CG	M	9.2	7.7	12.9
CG	F	7.3	7.7	9.2
CG	M	9.7	15.4	25.0
CG	F	7.5	38.5	29.2
CG	M	5.9	7.7	22.2
CG	M	8.1	7.7	16.7
CG	M	4.6	18.0	19.4
CG	M	12	7.7	13.9
CG	M	6.1	23.1	15.3
IG	F	7	1.0	8.3
IG	F	6.4	51.1	43.1
IG	M	11	7.7	8.3
IG	M	6.2	7.7	22.2
IG	F	6.8	7.7	18.1
IG	M	8	7.7	25.0
IG	F	5.8	18	22.2
IG	M	4.8	20.5	19.4
IG	M	7.9	25.6	25.0
IG	F	7.6	20.5	11.1

FG, follow group. CG, control group. IG, intervention group. F, female. M, male. GMFD, gross motor. Function standing. GMFE, gross motor function walking.
